# Electrical Conductivity of a Stretching Viscoelastic Filament

**DOI:** 10.3390/ma14051294

**Published:** 2021-03-08

**Authors:** Manuel Rubio, Samir Sadek, Emilio José Vega, Alfonso Miguel Gañán-Calvo, José María Montanero

**Affiliations:** 1 Departamento de Ingeniería Mecánica, Energética y de los Materiales and Instituto de Computación Científica Avanzada (ICCAEx), Universidad de Extremadura, Avda. de Elvas s/n, E-06071 Badajoz, Spain; marubio@unex.es (M.R.); sadek@unex.es (S.S.); ejvega@unex.es (E.J.V.); 2 Laboratory of Engineering for Energy and Environmental Sustainability, Department of Fluid Mechanics and Aerospace Engineering, University of Seville, E-41092 Seville, Spain; amgc@us.es

**Keywords:** electrical conductivity, viscoelastic filament, electrospinning

## Abstract

Long polymeric chains highly stretched and aligned with the flow confer a strong mechanical anisotropy on a viscoelastic solution. The electrically-driven transport of free ions under such conditions is far from being understood. In this paper, we determine experimentally whether the above-mentioned deviation from isotropy affects the electric charge transport across the liquid. To this end, we measure the electrical conductivity in the flow (stretching) direction of the cylindrical liquid filament formed in the elasto-capillary thinning that arises during the breakup of a viscoelastic liquid bridge. First, we examine the behavior of monodisperse solutions of polyethylene oxide (PEO) in a mixture of glycerine and water. For all the concentrations and molecular weights considered, the filament conductivity remains practically the same as the isotropic conductivity measured under hydrostatic conditions. However, we observe a decrease in the electric current at the end of elasto-capillary regime which may partially be attributed to the reduction of the liquid conductivity. Then, we measure the conductivity of bidisperse solutions of PEO with very different molecular weights. In this case, a significant decrease in conductivity is observed as the filament radius decreases. This constitutes the first experimental evidence of ion mobility reduction in stretching viscoelastic filaments, a relevant effect in applications such as electrospinning.

## 1. Introduction

Many applications involving liquid handling at microscales take advantage of the inherently clean action of electromagnetic fields. Assuming the most usual case where magnetic effects are negligible, electric fields drive the motion of ions normally present in liquid solvents. In most cases, solvents are molecularly simple, and the classical ohmic conduction model can be adopted to calculate the current density at any location. In this model, the liquid is assumed to be isotropic, and the proportion between the current density transported by ions and the electric field acting on them is a scalar conductivity which depends only on the liquid temperature.

In many microfluidic applications such as electrospinning [1,2,3], the presence of long polymeric chains, considerably stretched and highly aligned with the flow, confers a strong mechanical anisotropy on the viscoelastic solution. Thus, the extensional viscosity can reach values several orders of magnitude larger than the shear viscosity due to the growth of elastic stresses in the flow direction [4,5]. One may argue that if considerable non-Newtonian deviations arise in the stress-strain rate relationship, then a measurable departure from isotropy should appear in the rest of the transport properties too. In particular, the electrical conductivity $K_{\|}$ in the flow (stretching) direction may differ from that along the perpendicular axis, $K_{⊥}$. These two conductivities may in turn take values different from the conductivity $K_{0}$ measured under hydrostatic conditions, i.e., with the polymers at their equilibrium coiling state. If the dissolved ionic species is sufficiently small or electrolytes are used as solvents, isotropic electrical conduction may be expected even under high mechanical anisotropy. In any case, the electrically-driven transport of free ions when long polymeric chains are highly stretched and aligned with the flow is far from being understood, partly because the accurate measurement of the electrical conductivity under such conditions constitutes a difficult task.

**Figure 1:**
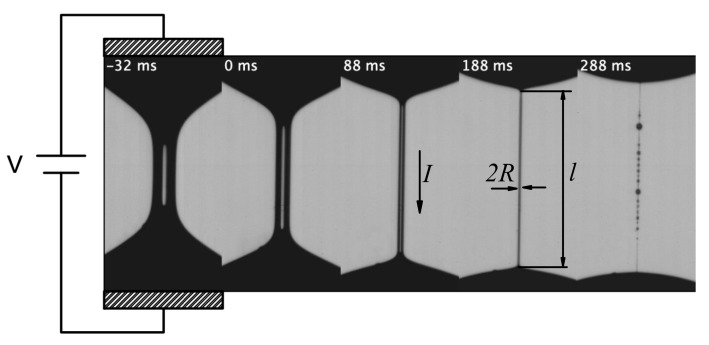
Sequence of experimental images showing the elasto-capillary thinning arising during the breakup of a liquid bridge of polyethylene oxide (PEO) with molecular weight $M_{w}=8\times 10^{6}$ g/mol dissolved in a mixture of deionized water and glycerine 50/50% (*w*/*w*) at a concentration of 1% by weight. The images show only the central part of the liquid bridge. The filament adopts a cylindrical shape of radius *R* and length *ℓ* from the instant t=0 to 188 ms. The shape deviates from the cylinder from t=188 ms due to the sequence of three effects: the shrinkage of the filament-droplet transition region, the beads-on-string instability, and the blistering instability.

In the elasto-capillary thinning emerging during the breakup of liquid bridges of polymer solutions [6], an essentially uniaxial extensional velocity field is generated within a quasi-cylindrical filament far away from the supporting disks [7] (Figure 1). The time evolution of the filament radius is given by the exponential function R(t)=Aexp[−t/(3λ)], where *A* is a constant and λ is the extensional relaxation time [8]. The filament thinning results in a natural (self-selected) stretching rate which exceeds the threshold leading to the so-called coil-stretch transition [6]. As a consequence, the polymer chains keep stretching during the thinning so that the growing elastic stress balances the increasing capillary pressure in the filament [9,10], and the extensional viscosity grows exponentially on time. This elasto-capillary regime eventually gives rise to the so-called beads-on-a-string (BOAS) [11] and blistering [12] instabilities for sufficiently elastic liquids.

The capillary breakup of a liquid bridge constitutes an excellent test bench to measure the conductivity in the flow (stretching) direction, $K_{\|}$, of a viscoelastic solution under different stretching conditions. The quasi-cylindrical filament formed in the elasto-capillary regime is a liquid “wire” connecting two equipotential drops (Figure 1). The microscopic structure of this wire continuously changes as the dissolved polymers stretch during the filament thinning. Thus, the parallel electrical conductivity $K_{\|}$ can readily be measured for different stretching conditions in a single experiment. The balance between elasticity and surface tension in the elasto-capillary regime establishes the fixed temporal scale λ over the whole filament, i.e., the relative variation of the filament radius, $R^{-1}\;\left|dR/dt\right|=1/\left(3\lambda \right)$, remains constant. This contrasts with what occurs in the pinch-off of a Newtonian liquid thread, where the temporal scale vanishes in the vicinity of the pinching point [13,14]. The temporal scale λ of the experiment can be adjusted by properly selecting the polymer concentration and molecular weight [9,15], which facilitates the measurement of the electric current and avoids undesirable effects, such as time variation of the electrical permittivity [16].

Polyethylene oxide (PEO) is a biologically inactive yet electroactive polymer, which has found an enormous range of applications from Pharmacy (including COVID-19 vaccines) and Biotechnology to lithium batteries. The PEO molecule contains both hydrophobic and hydrophilic sites with hydroxyl and hydrogen terminations at its two ends. These characteristics endow the molecule with complex behavior and the capacity to respond to numerous mechanical and physicochemical actions. For instance, high-molecular-weight PEO polymers can form relatively big, macroscopic spherulites susceptible to large mechanical deformations [17].

Rubio et al. [18] have recently analyzed both theoretically and experimentally the breakup of electrified liquid bridges of PEO dissolved in an aqueous solution of glycerine. The major motivation of that work was to describe the competition between hydrodynamic and Maxwell stresses, as well as the influence of the intense electric field on the extensional relaxation time. For that purpose, voltages of the order of some kilovolts were applied, which led to a significant increase in the liquid bridge temperature due to the Joule effect. In this work, we will focus on the experimental measurement of the liquid conductivity under isothermal conditions, which requires much lower voltages. Due to the low values of the applied voltages, Maxwell stresses have negligible effects on the filament dynamics. Our goal is to check whether the parallel electrical conductivity $K_{\|}$ of the thinning viscoelastic filament changes as its radius decreases (the polymer chain length increases).

## 2. Experimental Method

**Figure 2:**
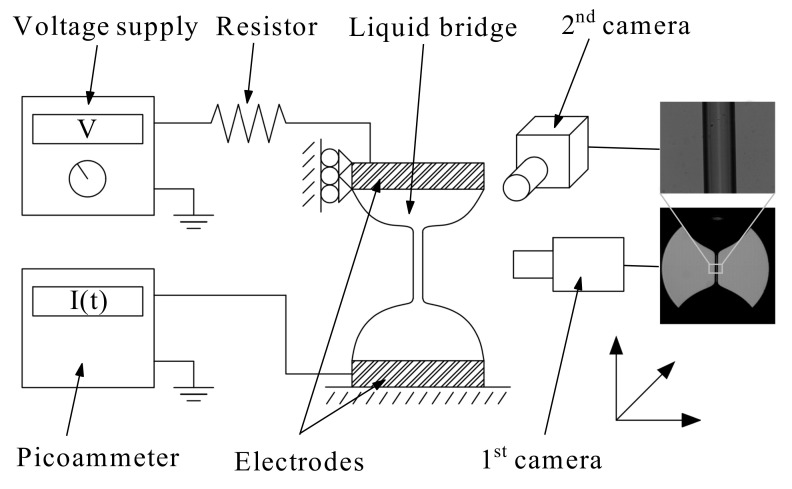
Sketch of the experimental configuration.

In our experiments, a viscoelastic liquid bridge was formed between two horizontal disks 1 mm in radius by injecting liquid with a syringe pump (KD Scientific, Legato 210 Series, Holliston, MA, USA) through an orifice 200 μm in diameter located in the center of the upper disk (Figure 2). The edges of the disks were sharpened so that the triple contact lines remained pinned to those edges during the experiment. The two disks were fixed to high-precision orientation systems to ensure their correct alignment. The upper disk was moved up with a vertical motorized stage (Z825B connected to KDC101, Thorlabs, Newton, NJ, USA) at a constant speed sufficiently small for the process to be quasi-static.

A direct current (DC) voltage drop was set between the upper and lower (grounded) disks using a power voltage supply (HQ Power PS3003, Weifang, China, 0–30 V). The electric potential was applied during the last phase of the liquid bridge quasi-static stretching to avoid the liquid heating produced by the Joule effect. In addition, a resistor of resistance $R_{r}=47$ MΩ was connected in series with the liquid bridge. This element reduces the electric current crossing the circuit before the elasto-capillary regime is reached. The electric current was measured with a picoammeter (Keithley model 6485, Cleveland, OH, USA).

Digital images of the liquid bridge were acquired with two synchronized high-speed cameras with optical axes perpendicular to each other (Figure 2). One of the cameras (Photron Fastcam SA5, Tokio, Japan) was equipped with a set of optical lenses (Optem Zoom 70 XL, Calgary, AB, Canada) with a variable magnification from 0.75× to 5.25× to measure the filament length formed during the elastocapillary regime. The magnification used was 2.75 μm/pixel. The other camera (Photron Fastcam Mini UX50,Tokio, Japan) was equipped with a set of optical lenses that consisted of a 10× magnification zoom-objective (Optem HR) and a system of lenses (Optem Zoom 70 XL) with a variable magnification from 0.75× to 5.25× to measure accurately the viscoelastic filament diameter. The magnification used was 0.19 μm/pixel. Images of 1024 × 1024 pixels were acquired with the two cameras at 125 fps by illuminating the liquid bridge from the back with cool white light provided by optical fibers connected to light sources. Frosted diffusers were positioned between the optical fibers and the liquid bridge to provide a uniformly lit background.

In the experiments, the liquid bridge was broken with the slow retraction method [15,19]. A liquid bridge of volume around 7 mm${}^{3}$ was formed between the supporting disks separated initially by a distance around 2 mm. The lower disk remained still. The liquid bridge was stretched by moving the upper disk away from the lower one at the speed v=0.2 mm/s. This speed corresponds to a Capillary number Ca$=\eta_{0}v/\gamma$ ($\eta_{0}$ is the zero-shear viscosity and γ the surface tension) smaller than 4 × 10${}^{-3}$, and, therefore, dynamical effects of the stretching process were expected to be small [20]. A voltage V=30 V was set right before the liquid bridge reached the stability limit. The images acquired in the experiments were processed with a sub-pixel resolution technique [21] to determine accurately the free surface position. The use of a pixel-resolution method becomes inaccurate as the filament radius decreases. We calculated the filament length ℓ(t) from the images acquired with the first camera mentioned above. This length was calculated as the distance between the two liquid bridge sections whose diameters were twice the minimum diameter. We checked that this choice does not significantly affect the results. The filament radius R(t) was measured from the images taken by the second camera mentioned above.

This liquid bridge configuration has been widely used as a test-bench to study the stretching of a viscoelastic filament. However, other configurations are possible for our study too. For instance, the dripping-onto-substrate configuration [22,23] would also allow one to produce the elasto-capillary thinning of a viscoelastic filament between two solid electrodes. However, the wetting on the lower substrate may affect the reproducibility of the results in terms of the thinning filament location, which may hinder the accurate measurement of the filament radius. The slow retraction method has been widely used for the determination of relaxation times in extensional rheometry experiments, reducing the dynamical effects of the stretching process. The use of other techniques such as CaBER rheometry [24] may produce undesirable effects for our purposes, such as an excessively large filament.

The fluids used in the experiments were solutions of PEO in a glycerin-water mixture 50/50% (*w*/*w*). We used deionized water for synthesis (Millipore, Sigma-Aldrich, Saint Louis, MO, USA) ) with a conductivity $4.3\times 10^{-4}$ S/m at 20 ${}^{\circ }$C, and glycerol, 99% for synthesis (PanReac AppliChem, ITW Reagents, Chicago, IL, USA). Glycerol of this high purity can be regarded as a quasi-dielectric liquid. In fact, its conductivity is smaller than $10^{-8}$ S/m, the minimum value measurable with our conductometer. The conductivity of the water-glycerol mixture was $8\times 10^{-5}$ S/m. In the first set of experiments, we examined the behavior of monodisperse viscoelastic solutions. In this case, the polymer molecular weight was either $M_{w}=2\times 10^{6}$ g/mol (PEO2M) or $8\times 10^{6}$ g/mol (PEO8M), and the polymers were dissolved at different concentrations (see Table 1). In the last set of experiments, we considered bidisperse solutions of PEO. First, we dissolved PEO8M at the concentration 0.5% (*w*/*w*), and then PEO with $M_{w}=10^{5}$ g/mol (PEO100K) at the concentrations 5%, 9.1% and 15% (*w*/*w*). We prepared stock solutions by dissolving the polymers in the solvent with a magnetic stirrer at low angular speeds to minimize the mechanical degradation of the long polymer chains. We took special care with the manipulation of our reagents to exclude degradation by contaminants, such as halide ions. As mentioned in the Introduction, PEO is an ideal candidate for our study. Nevertheless, we also examined the behavior of poly(acrylic acid) (PAA). In this case, the filament did not adopt a quasi-cylindrical shape over the elasto-capillary regime, which (as explained below) prevented us from accurately measuring the filament’s electrical conductivity. We also verified that the use of pure water as solvent produced the same undesirable effect.

**Figure 3:**
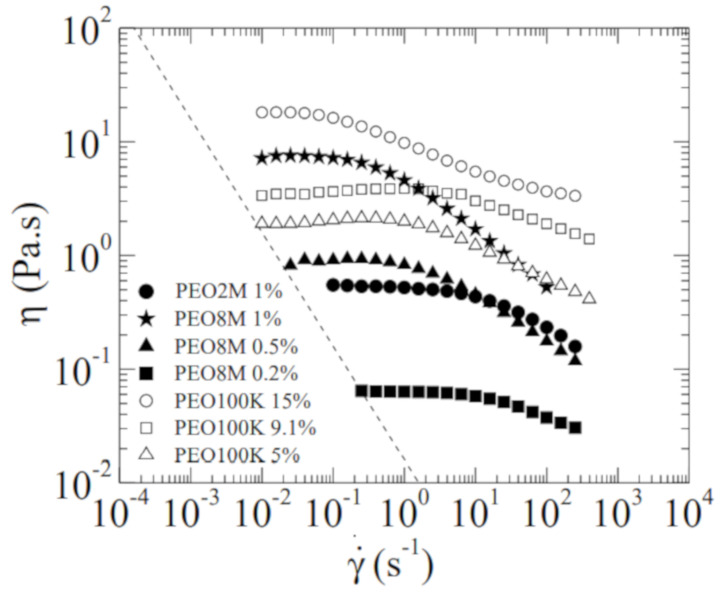
Dependence of the solution shear viscosity η upon the shear rate $\dot{\gamma }$ at 20 ${}^{\circ }$C. The dashed line corresponds to the low-shear-rate limit based on 20× the minimum resolvable torque of the shear rheometer used.

The density ρ of the tested fluids was measured with a pycnometer of 10±1 mL and a precision balance. The surface tension γ was measured with the Theoretical Interface Fitting Analysis (TIFA) method [25]. The extensional relaxation time λ was determined from the experiments because the mechanical effect of the electric field was negligible. The hydrostatic electrical conductivity $K_{0}$ was measured by applying a voltage difference between the ends of a borosilicate capillary filled with the tested liquid. The value of $K_{0}$ was determined from the slope of the linear relationship between the applied voltage and measured electric current. Table 1 shows the properties of the tested liquids. A stress-controlled rotational rheometer (Discovery HR-2, TA Instruments, New Castle, UK) was used to measure the steady shear viscosity η as a function of the shear rate $\dot{\gamma }$ of the fluid samples (Figure 3). We used a plate-plate geometry of 20 mm in radius and a gap of 1 mm. The temperature within the fluid volume was set to 20 ${}^{\circ }$C, and was controlled by a Peltier element. The zero-shear viscosity of the bidisperse solutions significantly increases when PEO100K is added to the solution PEO8M 0.5%. As will be shown in Section 2, this allows the filament to stretch down to R≃2 μm while keeping its quasi-cylindrical shape. The extensional relaxation time of the bidisperse solutions is significantly smaller than that of PEO8M 0.5% probably due to the interaction between polymers. This suggests that dissolved polymers with $M_{w}=8\times 10^{6}$ g/mol do not properly stretch in the presence of those with $M_{w}=10^{5}$ g/mol.

As mentioned above, a quasi-cylindrical liquid filament of radius R(t) and length ℓ(t) connected the upper and lower parent drops during the elasto-capillary regime in all the experiments analyzed in this work (Figure 1). We made sure that the filament did not shrink at its ends during the analyzed time interval so that no extra voltage drop took place in that region. The conductivity parallel to the flow of this liquid “wire” can readily be calculated as

(1) $$K_{\|}=\frac{I}{V-IR_{r}}\;\frac{\ell }{\pi R^{2}},$$

where I(t) is the electric current. We repeated several times the same experiment. In each experimental realization, we interpolated the conductivity data to obtain a continuous curve $K_{\|}\left(R\right)$. The results of $K_{\|}$ shown in Figures 5 and 7 are the average values among different realizations obtained from those curves at sampled instants, while the error bars are the corresponding standard deviations.

## 3. Results and Discussion

**Figure 4:**
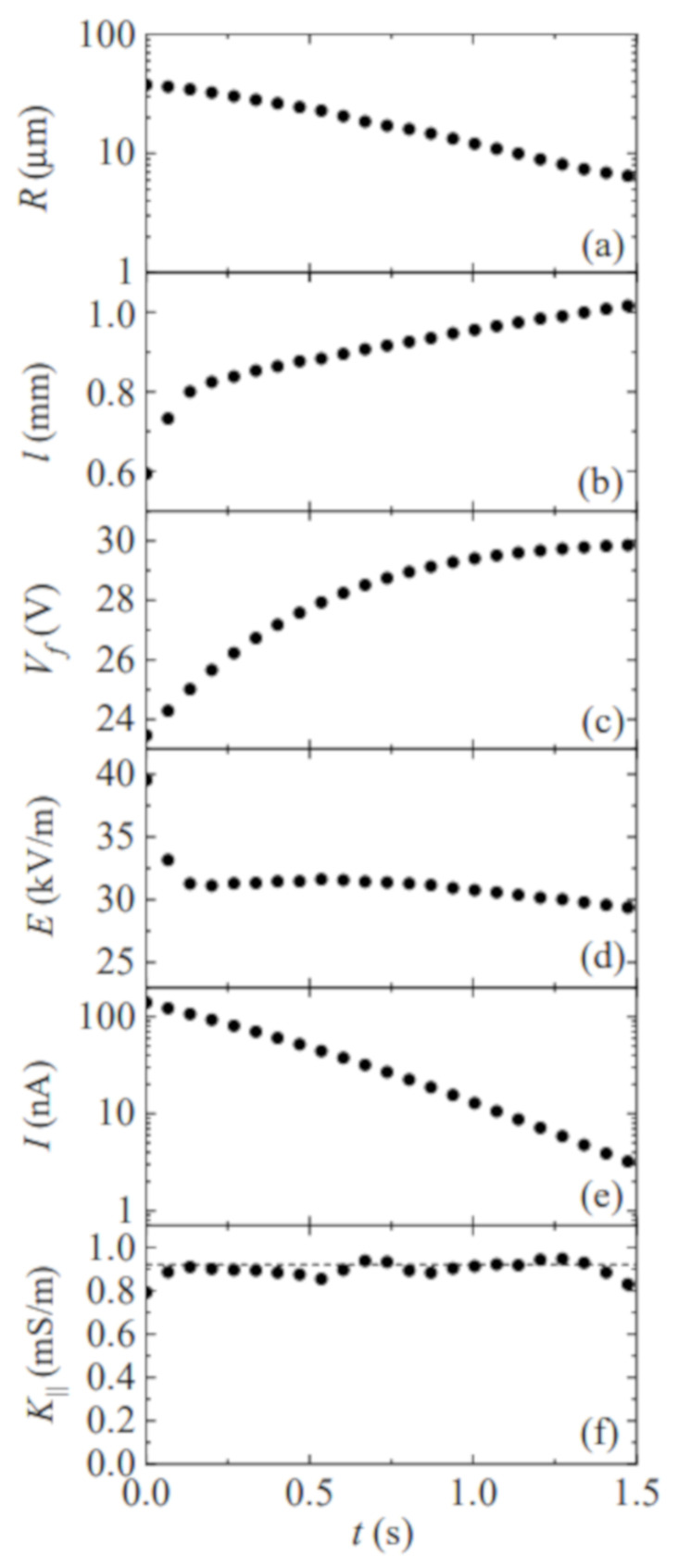
Filament radius *R* (**a**) and length *ℓ* (**b**), voltage drop across the filament $V_{f}$ (**c**), electric field $E=V_{f}/\ell$ (**d**), electric current *I* (**e**), and the conductivity $K_{\|}$ (**f**) as a function of time during the breakup of a liquid bridge of PEO8M 0.5%. The time origin t=0 s corresponds to the instant at which the picoammeter starts measuring for I≲200 nA. The dashed line in graph (**f**) is the value of the hydrostatic conductivity.

Figure 4 shows the filament radius *R* and length *ℓ*, voltage drop across the filament $V_{f}=V-IR_{r}$, electric field $E=V_{f}/\ell$, electric current *I*, and the conductivity $K_{\|}$ as a function of time during the breakup of a liquid bridge of PEO8M 0.5%. The electrical resistance of the resistor and liquid filament are commensurate with each other during the initial phase of the elasto-capillary regime. For this reason, the voltage drop across the filament significantly increases in that phase. As can be observed, the electrical conductivity is essentially independent of the instantaneous voltage drop. The filament length increases as the radius decreases. This effect partially compensates for the increase of $V_{f}$, and thus the electric field remains practically constant. In the last stage of the elasto-capillary regime, the free surface shrinks at the filament ends, which may significantly reduce the electric current. This deviation from the cylindrical shape assumed in Equation (1) does not allow us to measure accurately the electrical conductivity for R≲7–10 μm. Determining quantitatively the influence of the filament end shrinkage on the electric current would require the accurate measurement of the filament radius over the whole filament, which is not possible owing to the disparity between the radial and axial length scales.

**Figure 5:**
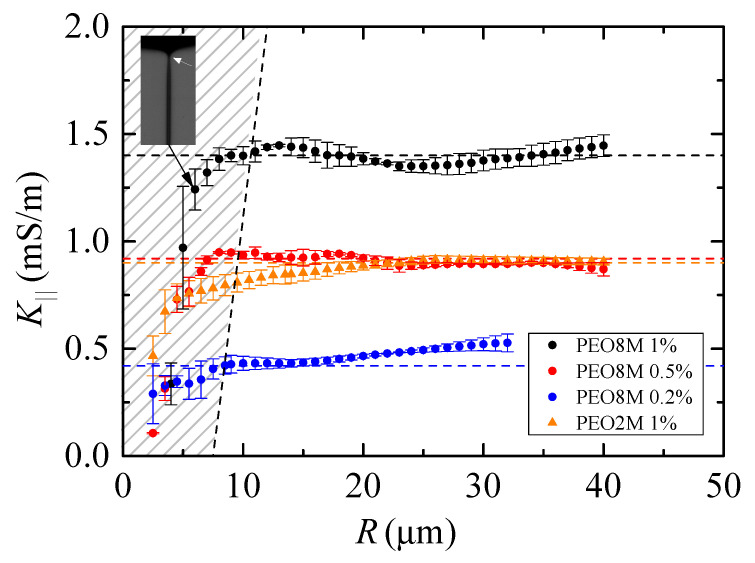
Electrical conductivity $K_{\|}$ as a function of the filament radius *R*. The symbols correspond to results obtained for different liquids, while the dashed lines are the hydrostatic values. The stripped area corresponds to values of *R* for which the filament shape significantly deviates from the cylinder due to the shrinkage of the filament ends. The inset shows the shrinkage of the filament end in one of the cases reported in the figure.

The results for the electrical conductivity $K_{\|}$ of the monodisperse polymeric solutions are shown in Figure 5. The wavy shape of $K_{\|}\left(R\right)$ is probably due to the criterion adopted to determine the filament length *ℓ*, which may produce small spurious variations of that quantity. The main conclusion is that the electrical conductivity is practically the same as that measured under hydrostatic conditions (Table 1) even though the dissolved polymers are highly stretched in the elasto-capillary regime. Unfortunately, and as mentioned above, we cannot obtain reliable results for R≲7−10 μm (the stripped area in Figure 5) because the filament shape deviates from the cylinder as *R* decreases below those values. Therefore, we cannot determine to what extent the decrease of $K_{\|}$ as calculated from (1) can be attributed to a true reduction of the liquid conductivity.

The results for the elasto-capillary regime shown in Figure 5 should not be necessarily expected. It must be noted that polymers occupy a significant hydrodynamic volume in all the cases analyzed even if they remained at their equilibrium coiling state. The number of macromolecules per unit liquid volume can be calculated as $n_{p}=c\;\rho /\left(M_{w}/N_{A}\right)$, where *c* is the mass polymer concentration, ρ is the solution density, $M_{w}$ is the molecular weight, and $N_{A}$ is the Avogadro number. The fraction of liquid volume occupied by the coiled polymers is $\hat{v}=V_{c}n_{p}$, where $V_{c}=4/3\;\pi R_{\mathrm{hyd}}^{3}$ is the effective volume of the polymer at the equilibrium coiling state, and $R_{\mathrm{hyd}}$ is the polymer hydrodynamic radius [26]. This radius can be replaced with the gyration radius $R_{g}$, although they take similar values. For PEO2M 1%, $\hat{v}=1.78$, which suggests that polymers at their coiling state occupy a very significant volume of the viscoelastic solution. In this case, $R_{\mathrm{hyd}}$ is an overestimation of the polymer effective radius due to the interaction among the macromolecules. In fact, the concentration is much higher than the overlap concentration $c^{*}$.

When the polymer is stretched, the effective volume $V_{c}$ can be replaced with $V_{s}=\pi R_{\mathrm{ef}}^{2}L_{p}$, where $R_{\mathrm{ef}}$ represents an effective radius and $L_{p}$ the polymeric chain length. This length takes values of the order of ${\left(L_{E}^{2}\right)}^{1/2}R_{g}$ [5] at the end of the elasto-capillary regime, where the maximum stretching has been reached. The finite extensibility parameter $L_{E}^{2}$ takes very large values (see Table 1), and therefore the liquid volume affected by the polymers may increase considerably at the end of the elasto-capillary regime. The fact that the electrical conductivity remains constant during the filament thinning may suggest that the polymer cross-section seen by the ions considerably decreases as the polymer stretches.

Walden’s rule [27] establishes that K·μ≃ const. for the same ions in different solvents or in the same solvent at different temperatures. This empirical rule can be rationalized in terms of the diffusion coefficient as follows. The Einstein relation establishes that the ion mobility, and therefore the electrical conductivity, is proportional to the diffusion coefficient of ions in the solvent. According to the Stokes–Einstein equation, the ion diffusion coefficient is inversely proportional to the solvent viscosity. Therefore, the electrical conductivity is inversely proportional to the solvent viscosity (Walden’s rule). In our experiments, Walden’s rule dramatically fails in the flow (electric field) direction. In fact, the extensional viscosity increases exponentially over time, while the electrical conductivity $K_{\|}$ remains practically constant. This occurs because the extensional viscosity is associated with the stretched polymers, while the electrical conductivity is related to the diffusion of ions through the solvent molecules, which does not seem to be significantly affected by the polymer stretching. In other words, (electrically-driven) diffusion of ions and diffusion of axial momentum seem to be independent transport phenomena because they are associated with very different molecules present in the viscoelastic solution.

**Figure 6:**
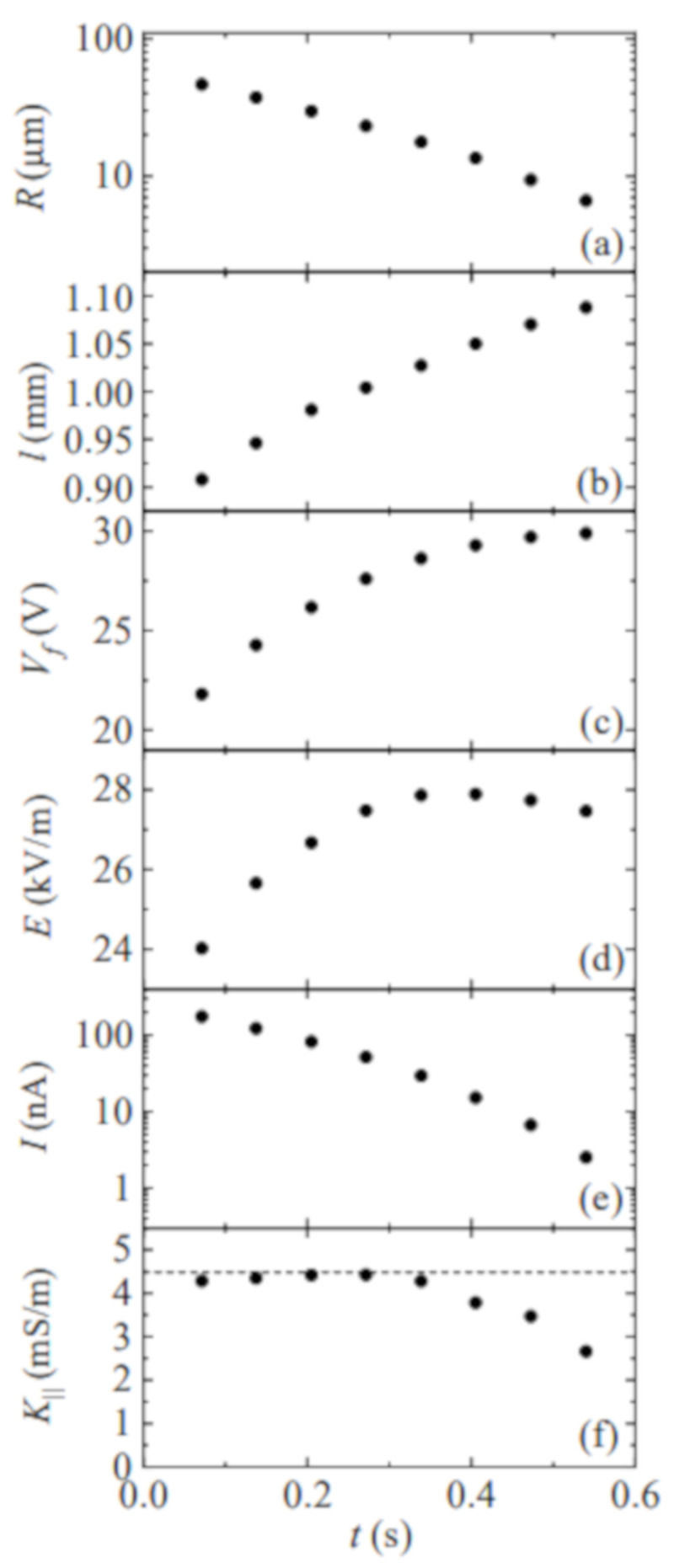
Filament radius *R* (**a**) and length *ℓ* (**b**), voltage drop across the filament $V_{f}$ (**c**), electric field $E=V_{f}/\ell$ (**d**), electric current *I* (**e**), and the conductivity $K_{\|}$ (**f**) as a function of time during the breakup of a liquid bridge of PEO100K 9.1%. The time origin t=0 s corresponds to the instant at which the picoammeter starts measuring for I≲200 nA. The dashed line in graph (**f**) is the value of the hydrostatic conductivity.

**Figure 7:**
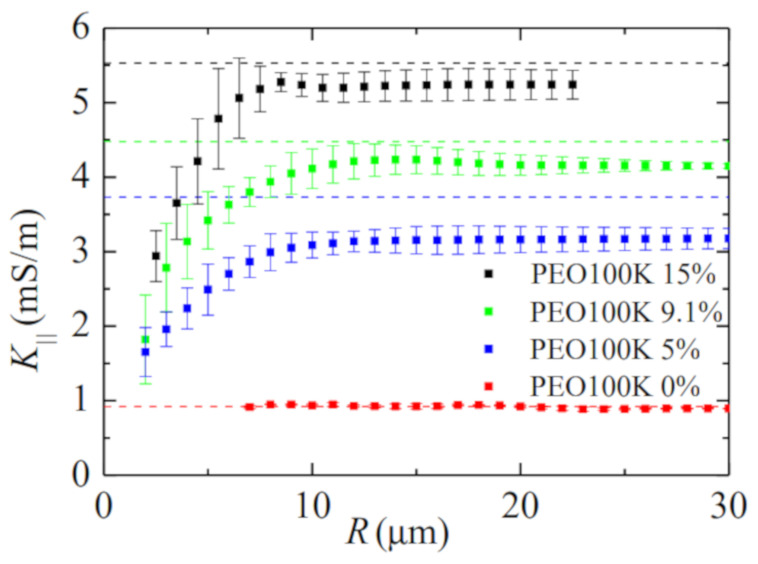
Electrical conductivity $K_{\|}$ as a function of the filament radius *R*. The symbols correspond to results obtained for different liquids, while the dashed lines are the hydrostatic values.

**Figure 8:**
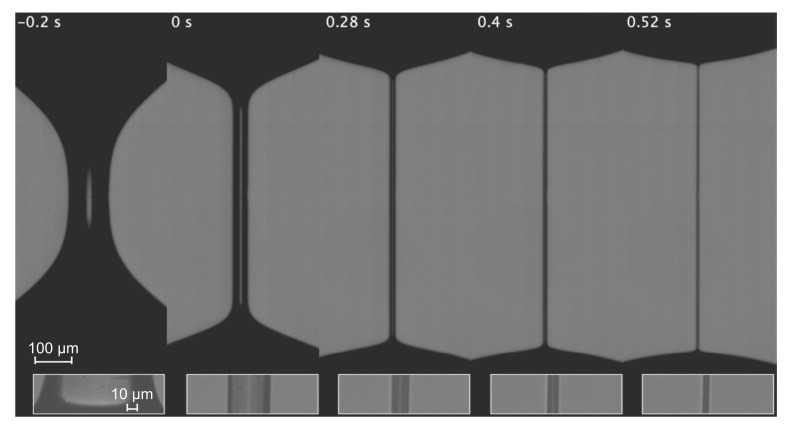
Images of the filament during the breakup of a liquid bridge of PEO100K 9.1%. The insets show the images of the center of the filament used to measure the filament radius. The instant t=0.52 s corresponds to the last point of $K_{\|}\left(R\right)$ in Figure 7.

In the second set of experiments, we measured the conductivity of bidisperse solutions of PEO with very different molecular weights. For the sake of illustration, Figure 6 shows the time evolution of the quantities of interest in a single experiment with PEO100K 9.1%. The extensional relaxation time characterizing the elasto-capillary regime is essentially determined by the polymer with the highest molecular weight. This means that the polymer molecules with the lowest molecular weight do not undergo the coil-to-stretch transition, and, therefore, they remain in their coiling state. As can be observed in Figure 7, the liquid conductivity considerably decreases as the filament radius decreases below around 7 μm. This effect becomes more noticeable as the concentration of PEO100K increases. As shown in Figure 8, this reduction of the electrical conductivity cannot be attributed to deviations of the filament shape from the cylindrical shape assumed in our calculations. In fact, the shrinkage of the filament ends takes place for R≲2 μm probably due to the higher solution shear viscosity (Table 1). There are small differences between the value of $K_{\|}$ and $K_{0}$ for large *R* when PEO100K is added to the solution. This is probably due to the early orientation of the polymer molecules in the axial direction as soon as a filament begins to form. We cannot ascertain if the reduction of the solution conductivity in Figure 8 for small radii is caused exclusively by the presence of coiled polymer molecules in the filament. It is also possible that the bigger polymers contribute to the reduction of the electrical conductivity, as suggested by the results in Figure 5.

## 4. Concluding Remarks

We measured the electrical conductivity in the stretching direction of thinning viscoelastic filaments of PEO dissolved in a mixture of glycerine and water. The conductivity of monodisperse solutions of polymers with high molecular weights remained practically the same as that measured under hydrostatic conditions for filament radii *R* larger than about 7–10 μm. Nevertheless, we observed a decrease of the electric current at the end of the elasto-capillary thinning which may partially be caused by the reduction of the liquid conductivity. However, the shrinkage of the filament ends in that time interval prevented us from coming to a reliable conclusion. Adding polymers with a low molecular weight at a high concentration allowed us to measure the conductivity for radius down to R≃2 μm. A significant decrease in the conductivity was observed for these bidisperse solutions in the range 2≲R≲7 μm.

Our results constitute the first experimental evidence of ion mobility reduction in thin viscoelastic filaments, an important effect in applications such as electrospinning. In this technique, viscoelastic filaments with diameters much smaller than those analyzed in our study are extruded under the action of an externally applied electric field. Therefore, the reduction of the electrical conductivity is expected to be much more noticeable in that case.

As mentioned in the Introduction, isotropic bulk conductivity is the simplest model of charge transport owing to dissolved ions. However, the formation of diffuse electrical double (Debye) layers near the free surface can significantly affect charge transport in that region. On a macroscopic scale, these surface electrokinetic effects are typically accounted for with a surface conductivity [16,28,29]. Nevertheless, and for the electrical conductivities $K_{0}∼10^{-3}$ S/m considered in our experiments, the Debye layer thickness is expected to be much smaller than the filament radius [30], and, therefore, surface electrokinetic effects are expected to be negligible.

Our experiments allow us to measure the electrical conductivity $K_{\|}$, which characterizes the charge flux in the stretching direction. We could not measure the conductivity along the perpendicular (radial) direction, and, therefore, we cannot ensure whether anisotropy effects arise in the liquid filament due to the polymer stretching. The leaky-dielectric model, commonly used to analyze electrohydrodynamic phenomena in viscoelastic solutions, relies on the isotropic ohmic conduction approximation. Therefore, the failure of this approximation may invalidate to some extent the leaky-dielectric model predictions.

While it has been shown that the presence of macromolecules in electrolyte solutions decreases the electrical conductivity [31], the effects of PEO on aqueous solutions of organic non-dissociable polar molecules, such as glycerol, are less known. This research belongs to an intermediate realm between that of the simpler effects of long polar polymers on electrolytes [31] and that related to the anisotropic conductivity of biological tissues [32], which is of great applied interest. Mechanical and electrochemical effects, such as molecule elongation and formation of double layers, favor ionic channels among stretched macromolecules in polar solvents and solutions. This phenomenon requires intense scrutiny to which we aim to contribute. Our observations need further and deeper understanding for developing a physical model. For example, the relationship between the dipole moments of the molecules used in our study (see, e.g., Yamaguchi and Sato [33]) and the observed changes in the conductivity is the subject of ongoing studies.

## Figures and Tables

**Table 1:** Density ρ, zero-shear viscosity $\mu_{0}$, surface tension γ, extensional relaxation time λ, (hydrostatic) electrical conductivity $K_{0}$, overlap concentration $c^{*}$, hydrodynamic radius $R_{hyd}$, and finite extensibility parameter $L_{E}^{2}$ of the tested liquids at 20 ± 2 ${}^{\circ }$C.

Liquid	ρ (kg/m${}^{3}$)	$\mu_{0}$ (Pa·s)	γ (mN/m)	λ (ms)	$K_{0}$ (mS/m)	$c^{*}$ (wt%)	$R_{\mathrm{hyd}}$ (nm)	$L_{E}^{2}$
PEO2M 1%	1124.1±0.1	0.545	55±1	48±5	0.9±0	0.068	56.5	50,104
PEO8M 0.2%	1123.9±0.1	0.064	50±1	71±10	0.42±0.01	0.028	124.8	110,058
PEO8M 0.5%	1123.9±0.1	0.892	50±1	240±13	0.92±0.03	0.028	124.8	110,058
PEO8M 1%	1123.9±0.1	7.550	50±1	411±22	1.42±0.05	0.028	124.8	110,058
PEO100K 5%	1126.4	2.012	48±1	76±15	3.73±0.03	-	-	-
PEO100K 9.1%	1130.0	3.641	48±1	93±15	4.48±0.2	-	-	-
PEO100K 15%	1133.4	18.147	48±1	82±15	5.53±0.03	-	-	-

## Data Availability

Data sharing is not applicable to this article.
